# Diagnosis and management of cerebral folate deficiency: A form of folinic acid-responsive seizures

**Published:** 2014-10

**Authors:** Raidah S. Al-Baradie, Mohammed W. Chudary

**Affiliations:** *From the Department of Pediatric Neurology, King Fahd Specialist Hospital, Dammam, Kingdom of Saudi Arabia*

## Abstract

Folinic acid-responsive seizures (FARS) are a rare treatable cause of neonatal epilepsy. They have characteristic peaks on CSF monoamine metabolite analysis, and have mutations in the ALDH7A1 gene, characteristically found in pyridoxine-dependent epilepsy. There are case reports of patients presenting with seizures at a later age, and with folate deficiency due to different mechanisms with variable response to folinic acid supplementation. Here, we report 2 siblings who presented with global developmental delay and intractable seizures who responded clinically to folinic acid therapy. Their work-up included metabolic and genetic testing. The DNA sequencing was carried out for the ALDH7A1 gene, and the folate receptor 1 (FOLR1) gene. They had very low 5-methyltetrahydrofolate (5-MTHF) in CSF with no systemic folate deficiency and no characteristic peaks on neurotransmitter metabolite chromatogram. A novel mutation in the FOLR1 gene was found. The mutation in this gene is shown to affect CSF folate transport leading to cerebral folate deficiency. The response to treatment with folinic acid was dramatic with improvement in social interaction, mobility, and complete seizure control. We should consider the possibility of this treatable condition in appropriate clinical circumstances early, as diagnosis with favorable outcome depends on the specialized tests.

Folinic acid-responsive seizures (FARS) are a well-recognized cause of vitamin-responsive neonatal epilepsy.^1^ Patients present with seizures, either myoclonic or clonic, apnea, and irritability within 5 days after birth. Neuroimaging shows brain atrophy and variable white matter abnormalities. Some patients respond to pyridoxine alone, or have an initial response to it, only to become resistant later on and responding to folinic acid. The response to treatment has been variable. Gallagher et al^2^ reported that all surviving patients were developmentally delayed and 5 of 10 reported cases were deceased. Subsequently, it was demonstrated that FARS and pyridoxine-dependent epilepsy (PDE) are allelic; indeed, the molecular genetic basis of the 2 conditions is identical in at least some cases. Cerebrospinal fluid 5-methyltetrahydrofolate (5-MTHF) analysis was carried out in one of 7 cases, and reported normal in Hyland & Arnold’s study.^1^ A few cases of cerebral folate deficiency (CFD) presenting with seizures onset beyond the neonatal period (7 months to 11 years of age) have recently been reported with severe developmental regression,^3^ and movement disturbances with chorea,^4^ dystonia, and difficult ambulation.^3^,^4^ Neuroimaging showed brain atrophy and variable white matter abnormalities.^3^,^4^ The CSF biogenic metabolite did not show characteristic peaks, CSF 5-MTHF levels were found low,^4^ and FOLR1 gene mutations were reported.^3^,^4^ Mutations in the FOLR1 gene are associated with severely reduced concentrations of CSF 5-MTHF indicating that its gene product, folate receptor alpha (FRα), plays a crucial role in this transport process. This FRα defect causes a cerebral folate transport deficiency (MIM 136430), a progressive neurological disorder of late infantile onset that is characterized by psychomotor regression, epilepsy, and disturbed brain myelination as well as a depletion of white matter choline and often inositol.^3^ All of the cases responded to folinic acid supplementation becoming seizure free, or with better seizure control, and disappearance of chorea, and dystonia with better ambulation.^3^,^4^ Our objective in presenting these cases is to highlight that FARS is not uncommon. Early diagnosis and management can lead to better outcome.

## Case Report

Two siblings born to Saudi parents with first degree consanguinity at term through normal vaginal delivery, the boy weighing 2.6kg (second in birth order), and the girl weighing 2.5kg (third in birth order) had uneventful pregnancy, delivery, and early neonatal period. Both presented together to our services for intractable seizures. Two other siblings were healthy with no history of similar or other undiagnosed medical condition.

### Patient 1

**Figure 1:**
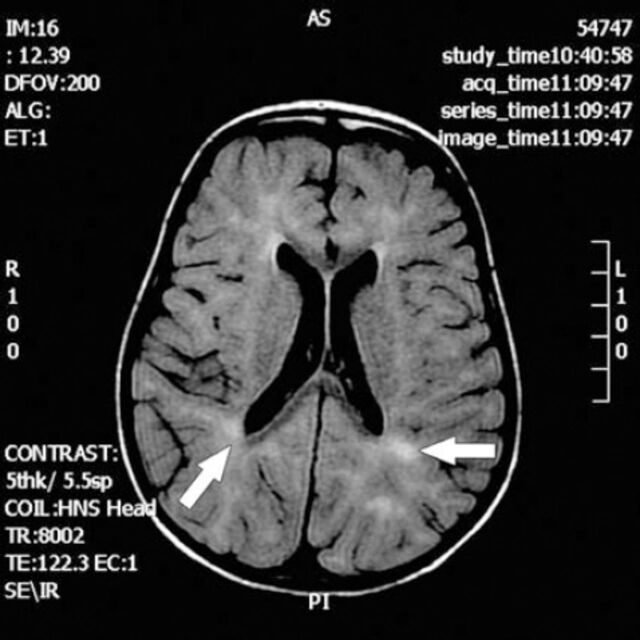
Magnetic resonance imaging (Flair) of a 5 years and 8 month-old male patient with cerebral folate deficiency showing normal myelin signal of callosal structures with decreased myelin signals for white matter and loss of myelin signals around occipital horns.

**Figure 2:**
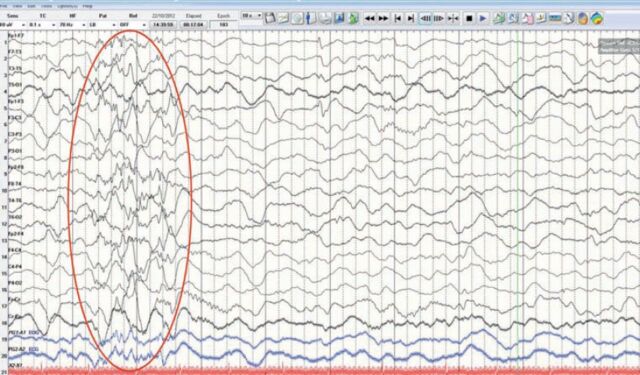
Electroencephalography (EEG) showed generalized slowing and generalized irregular epileptic discharges.

**Figure 3:**
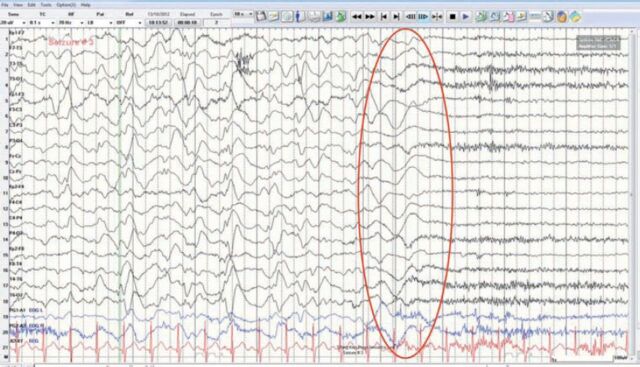
Electroencephalography (EEG) showed generalized slowing and multifocal spikes and sharp waves. In the second half of the EEG when patient developed drop attack (sudden fall), it correlates with generalized slow waves followed by generalized attenuation of the EEG.

The patient presented at the age of 5 years and 8 months with intractable seizures for the previous 15 months despite adequate doses of valproate (30 mg/kg/day) and vigabatrin (50 mg/kg/day). Seizures were in the form of drop attacks and myoclonic jerks, 3-5 episodes per day, mostly before going to sleep or after getting up. He had global developmental delay and rolled over at 9 months, sat at 12 months, crawled at 14 months, walked at 2 years, had vocabulary of only 30 words, and was not toilet trained. His symptoms were consistent with an autistic spectrum disorder. The neurological evaluation revealed generalized hypertonia, hyperreflexia, down going planters with unsteady wide-based gait with no neuro-cutaneous stigmata, with a head circumference of 51 cm. Systemic examination was unremarkable. Hearing and ophthalmological examination was normal. The brain MRI revealed sub cortical white matter abnormal high intensity consistent with hypomyelination with cerebellar atrophy (**Figure 1**). The electroencephalography (EEG) showed hypsarrhythmia initially changing one year later to generalized slowing and multifocal epileptic discharges, with an occasional attenuation of the EEG activity, which correlated with clinical drop attacks (**Figures 2 & 3**). Extensive metabolic work-up before starting empirical therapy including blood for ammonia, lactate, pyruvate, acylcarnitine, fatty acid profile, amino acids, biotinidase and urine for amino acids, organic acid, with CSF for glucose, lactate, pyruvate, and amino acids were normal. The serum folate was also normal (30.7 nmol/L, normal: 12-33). The CSF 5-MTHF was low (7 nmol/l, normal: 40-128), but neopterin, tetrahydrobiopterin (BH4), 5-hydroxyindolaceticacid (5-HIAA), homovanillic acid (HVA), 3-O-methyl dopa (3-O-MD) were normal, and did not show the characteristic peaks of FARS. He was started on pyridoxine, with folinic acid (0.75 mg/kg/day), which was gradually increased to the present dose of 1.7 mg/kg/day. At this dose of folinic acid he is free from seizures for the last 10 months, has marked improvement in his gait and social interaction, and EEG. He had breakthrough seizures once earlier when he ran out of his supplies of folinic acid for a 2-week period. The DNA analysis for ALDH7A1 gene mutation was normal, rather a homozygous novel mutation c.398C>A (p.Pro133His) in the FOLR1 gene was found. Analysis on prediction software (Polyphen-2 and Mutation Taster) (GCG Genetics, Lisbon, Portugal) indicates that it is probably pathogenic.

### Patient 2

This younger sister of patient one presented with drops attacks starting at the age of 4 years, which stopped with valproate for a few months, only to recur despite continued medication. Seizure semiology, frequency, and delayed development were similar to her brother. Neurological evaluation and EEG findings were the same. She had mild myopia, but normal hearing with a head circumference of 47 cm (between the third and fifteenth percentile for age). The brain MRI revealed increased signal intensity in sub cortical white matter, putamen, and head of caudate nucleus, and sub insular and anterior limbs of internal capsule hypomyelination with cerebellar atrophy. The MR spectroscopy was normal. All of the metabolic work-up was unremarkable except for the CSF 5-MTHF levels of 11 nmol/L (normal=40-150). She was started on pyridoxine (6mg/kg/day), and folinic acid like her brother, which was increased to 2mg/kg/day. She had dramatic EEG and neurological improvement like her brother. The DNA analysis for a ALDH7A1 gene mutation was normal, and she had the same homozygous mutation c.398C>A (p.Pro133His) in the FOLR1 gene, identical to her brother. Parents’ genetic screening was not obtained. Both patients were followed for 24 months after starting them on folinic acid. They stopped seizing, and their development improved markedly. They are going to special education schools, and are noticed to have better receptive and expressive language.

## Discussion

We report 2 siblings from the Middle East with CFD. This autosomal recessive disorder manifests in late infancy with severe developmental regression, movements disturbances, epilepsy, and leukodystrophy with low CSF 5-MTHF (the active folate metabolite) in the presence of normal folate metabolism outside the CNS.^3^ Folinic acid therapy can reverse the clinical symptoms and improve brain abnormalities and function.^3^ Mutations in the FOLR1 gene coding for folate receptor alpha (FRα) predominantly affecting cerebral folate transport leads to low CSF 5-MTHF levels. The choroid plexus is rich in FRα, and is the main site of folate delivery to the brain.

To date 10 cases have been reported in the literature with a mutation in the FOLR1 gene. These are missense (the most common mutation), nonsense, splice mutation, or duplication. These mutations lead to decreased protein expression, and/or loss of surface localization, mistargeting to intracellular compartments and thus absence of cellular binding of folic acid. These patients were Finnish, German, Italian, Turkish, Azerbaijani Gambian background with a high percentage of consanguineous marriages. All the 10 cases had ‘extremely low’ CSF 5-MTHF levels of ≤5 nmol/l. Other 4 cases with similar levels were found to have no mutation in FOLR1 or other folate transport genes. All of them with FOLR1 mutation presented initial with symptoms such as developmental delay, ataxia, and seizures within the first 3 years of life, and most of them developed motor deficits as well as behavioral abnormalities including autistic behavior. Apart from slow background and multifocal epileptiform activity in the EEG, most showed delayed myelination or hypomyelination of the cerebral white matter with pronounced cerebellar atrophy.^5^

Our siblings had a similar presentation, but the CSF 5-MTHF levels were slightly higher (7 and 11 nmol/l versus ≤5 nmol/l). They did not have low choline or low inositol as reported earlier in 4 out of 5, and 2 out of 5 cases.^5^ The lower the CSF 5-MTHF level, the higher are the chances of having a FOLR1 gene mutation, as 71% (10 out of 14) with levels ≤5 nmol/l had it, but it should still be considered in compatible clinical and radiological settings as seen with our cases. The CFD has also been linked to the presence of autoantibodies against the folate receptor in CSF,^6^ though most of these cases were earlier described as ‘idiopathic CFD’ earlier.

Secondary forms of CFD have been recognized during chronic use of antifolate and anticonvulsant drugs, and various known conditions such as Rett syndrome,^7^ dihydropteridine reductase deficiency, and Kearns-Sayre syndrome.^8^ Deficiency of dihydrofolate reductase, the enzyme responsible for catalyzing the conversion of dihydrofolate to tetrahydrofolate, causes cerebral folate deficiency with generalized tonic-clonic and focal seizures and megaloblastic anemia or pancytopenia.^9^ The CSF 5-MTHF levels tend to be less profoundly low in these cases as well as in those associated with folate receptor antibodies (FRA).

Cerebral folate deficiency has been shown to be strongly associated with autism or autistic spectrum disorders (ASD), the features were seen in our cases as well. In one large series of 93 ASD patients, all 16 CSF 5-MTHF values were below the normative mean CSF concentration, 75.3% of all 93 patients had FRα auto antibodies (FRA) in the serum samples, and an inverse relationship between the blocking FRA and CSF 5-MTHF was found. Approximately one-third of these, treated with folinic acid demonstrated moderate to much improvement in this study.^10^

The 5-MTHF is the precursor of the methyl-group donor S-adenosylmethionine, which is used in more than 100 chemical reactions. The methylation of arginine at position 107 within myelin basic protein is necessary to maintain stability of CNS myelin, and this might explain the hypomyelination seen in our cases. Biosynthesis of tetrahydrobiopterin is also dependent on 5-MTHF,^5^ and low levels of the later might be associated with low 5-HIAA, and HVA concentrations. We did not find low levels of these 2 in our patients, as with other cases.^3^,^8^ Our patients did not show the reduced choline and inositol peak on brain MRS as previously seen in some cases.^3^,^5^

In conclusion, patients presenting with intractable seizures beyond the neonatal period, especially in the setting of otherwise, unexplained global developmental delay or of features consistent with autistic spectrum disorder calls for consideration of cerebral folate deficiency. As only a limited number of cases are reported yet, more cases in the future will help us study the phenotype and better characterization of this new clinical entity.
